# Dyslipidemia Associated with Hypertension Increases the Risks for Coronary Heart Disease: A Case-Control Study in Harapan Kita Hospital, National Cardiovascular Center, Jakarta

**DOI:** 10.1155/2019/2517013

**Published:** 2019-04-30

**Authors:** Rea Ariyanti, Besral Besral

**Affiliations:** ^1^Faculty of Public Health, Universitas Indonesia, Kampus Baru FKM UI Depok 16424, Indonesia; ^2^Department of Biostatistics and Population Studies, Faculty of Public Health, Universitas Indonesia, Kampus Baru FKM UI Depok 16424, Indonesia

## Abstract

*Background. *Coronary Heart Disease (CHD) is the main highlight of the major cardiovascular diseases. In Indonesia, CHD is the leading cause of death from all deaths, with rates reaching 26.4%, which is four times greater than cancer mortality rates.* Objective.* This study aims to determine whether dyslipidemia associated with hypertension increases the risks for the incidence of CHD in Harapan Kita Hospital, National Cardiovascular Center, Jakarta, or does not.* Methods.* The study design was case control. The sample was 163 respondents, 82 respondents in the case group and 81 respondents in the control group. The data were analyzed by using logistic regression.* Results.* In the CHD group, the percentage of respondents with dyslipidemia was 50%, while in the control group, the percentage of respondents with dyslipidemia was 17.3%. The relationship of dyslipidemia with the incidence of CHD differed according to hypertension status. After being controlled for age, in hypertensive respondents, those with dyslipidemia was 18.1 times more likely to develop CHD compared with those nondyslipidemic, whereas in nonhypertensive respondents, those with dyslipidemia was 2.5 times more likely to develop CHD compared with those nondyslipidemic.* Recommendation.* It is recommended that the community have medical checkup regularly and change lifestyles by taking healthy diet to control lipid profile and blood pressure.

## 1. Introduction

Coronary Heart Disease (CHD) is one of the largest contributors to mortality and morbidity worldwide. Globally, CHD accounts for 17.5 million deaths in 2012, with over 75% of deaths occurring in developing countries [1, 2]. By 2015, 16% of all female and male deaths were caused by CHD [3]. In Indonesia, CHD is the leading cause of death from all deaths, with rates reaching 26.4%, which is four times higher than cancer death rates [4]. Basic Health Research (Riskesdas) in 2013 showed that the prevalence of CHD in Indonesia was 0.5% based on doctor-diagnosed interviews and 1.5% based on physicians' diagnosis with symptoms similar to CHD [5].

Broadly speaking, the cause of CHD is multifactorial in which some of them can be modified [6]. One of the modifiable risk factors is dyslipidemia. Dyslipidemia is defined as a lipid metabolic disorder characterized by an increase or decrease in lipid fraction in plasma [7]. Low-density lipoprotein cholesterol, and triglyceride, and low levels of high-density lipoprotein cholesterol are major risk factors of atherosclerosis affecting arteries of large and medium size and consequently causing ischemia in the heart [8].

Dyslipidemia is thought to be a primary risk factor for CHD and may play a role before other risk factors appear [9]. Dyslipidemia in Indonesia currently has a high prevalence rate. The prevalence of dyslipidemia based on Riskesdas Report of Biomedical Field in 2007 was 39.8% when viewed from total cholesterol >200 mg/dl. Report of Riskesdas in 2013 showed that there are 35.9% of Indonesian population aged ≥15 years with cholesterol levels [3].

Harapan Kita Hospital, National Cardiovascular Center, is a special hospital which is the National Referral Center for handling of heart and blood vessel disease. In addition, Harapan Kita Hospital is also one of the existing hospitals in Indonesia that serves as a Center for Cardiovascular Training and Education as well as a Center for Cardiovascular Research. Based on data from Harapan Kita Hospital, as many as 144,820 patients with heart and vascular disease (cardiovascular) came to visit in 2012. Of the total number of patients, most cases or about 3000 cases are coronary heart disease, as many as 2500 CHD patients without surgery and the remaining with surgery. This study aims to determine the relationship between dyslipidemia and the incidence of coronary heart disease in Harapan Kita Hospital year 2017. The novelty in this study is a factor dyslipidemia in this research assessed on three aspects such as HDL, LDL, and Triglyceride. This is different from previous study, where dyslipidemia just was assessed based on one of the three aspects.

## 2. Methods

This case-control study used secondary data from medical record data from Harapan Kita Hospital. The weakness design of the study is susceptible to selection bias. To reduce the risk of selection bias in this study is use of simple randomization. The dependent variable was incidence of coronary heart disease (CHD), and the main independent variable was dyslipidemia status. The potential confounding variables were age, gender, family history of CHD, smoking habit, hypertension or history of hypertension, diabetes or history of diabetes, and obesity.

The population in this study was all patients in Harapan Kita Hospital. The sample in this study was patients who visited in January 2016 until December 2017. The sample of case group was patients diagnosed with CHD by the doctor, randomly selected a total of 82 respondents, while the control group sample was a patient diagnosed with Atrial Fibrillation and Flutter (AFF) by a physician, randomly selected 81 respondents. Ethical approval number from the hospital is LB.02.01/VII/222/KEP.065/2017.

The data were analyzed using a binomial regression statistic test where an interaction assessment and confounding test were conducted. The interaction between dyslipidemia status variable and potential confounding variables was assessed using the forward method, in which the interaction variables were entered one by one into logistic regression model. Variables were considered to interact if they had a* p* value < 0.05. The assessment of confounders was done by removing candidate confounding variables one by one, starting from the variable with the highest Wald* p* value. If the variable after being issued from the model caused on* odds ratio* (OR) of dyslipidemia status variable change greater than 10%, the variable was considered a confounder and remained in the model.

## 3. Results

In Table 1, the data showed the following: the percentage of respondents with CHD who mostly suffered from dyslipidemia (50%), aged <60 years (67.1%), male (74.4%), did not have family history of CHD (75.6%), did not have smoking habit (53.7%), did not have hypertension or hypertension history (62.2%), having diabetes or history of diabetes (53.7%), and nonobese (62.2%).

The results showed that respondents with dyslipidemia had odds to suffer CHD 4.8 times higher compared with nondyslipidemic respondents. Respondents aged ≥ 60 years had odds to suffer CHD 1.4 times higher than respondents <60 years old. Male respondents had odds to suffer CHD 3.5 times higher compared to female respondents. Respondents who had family history of CHD had odds to have CHD 2.1 times higher compared with respondents who did not have family history of CHD. Respondents who have smoking habit had odds to suffer CHD 1.7 times higher compared to respondents who do not have smoking habit. Respondents who were hypertensive or have history of hypertension had odds to suffer CHD 1.9 times higher compared to nonhypertensive respondents. Respondents with diabetes or history of diabetes had odds to suffer CHD 1.3 times higher compared with nondiabetic respondents. Furthermore, respondents with obesity had odds to suffer CHD 0.9 times lower compared to nonobese respondents.

In Table 2, the results showed that the mean of age in CHD group was older than non-CHD group, which was 56.5 years with standard deviation of 9.5, where the youngest age was 37 years and the oldest was 83 years. The mean of SBP in CHD group was higher than non-CHD group, which was 126.2 mmhg with standard deviation of 21.0, where the lowest SBP was 100 mmhg and the highest was 200 mmhg. The average of DBP in CHD group was higher than non-CHD group, which was 85.6 mmhg with standard deviation of 15.5, where the lowest DBP was 60 mmhg and the highest was 120 mmhg

Furthermore, before the multivariable analysis, stratification tests were conducted to determine the effect of a control variable on the main variables, i.e., dyslipidemia and CHD. In Table 3, full model of dyslipidemia with CHD (hierarchically well-formulated model), it appears that the interaction between dyslipidemia and hypertension has* p* value = 0.038.

In Table 4, final model of dyslipidemia connection with CHD shows that there is interaction between dyslipidemia and hypertension and age was found as confounder.

Table 5 shows the relationship of dyslipidemia with CHD according to hypertension. Once controlled for age, in hypertensive respondents or having history of hypertension, those with dyslipidemia were 18 times more likely to develop CHD compared to those nondyslipidemic. Whereas in respondents who were not hypertensive or have no history of hypertension, those with dyslipidemia had chance 2.5 times higher to suffer CHD than those nondyslipidemic.

## 4. Discussion

The results showed that in CHD group, the percentage of respondents with dyslipidemia was 50%, while in the non-CHD group, the percentage of respondents with dyslipidemia was 17.3%. The results of this study are in accordance with the results of previous studies [10]. Dyslipidemia is considered to have an important role in cardiovascular events, especially CHD. Dyslipidemia is investigated as a predictor of CHD; it has a role in the process of atherogenesis [11, 12].

The results showed that the relationship of dyslipidemia and the incidence of CHD was different according to hypertension status. At the same age, respondents with hypertension or history of hypertension and dyslipidemia had 18 times higher to develop CHD than nondyslipidemic respondents, whereas in nonhypertensive patients, respondents with dyslipidemia were 2.5 times higher to develop CHD compared to nondyslipidemic respondents. This study is in line with previous studies that stated that dyslipidemia interacts with high blood pressure (hypertension) in causing CHD [13, 14].

The risk of CHD in patients with dyslipidemia will increase if dyslipidemia is accompanied by one or more other CHD risk factors [15]. Various studies showed that other factors that can cause cardiovascular disease as hypertension. Dyslipidemia and hypertension are established risk factors of prime importance in cardiovascular disease [16]. If these two factors (dyslipidemia and hypertension) are present together, this will accelerate the process of atherosclerosis, thus increasing the risk of CHD. In Indonesia, people with hypertension are estimated at 15 million, but only 4% are controlled hypertension. Controlled hypertension means they suffer from hypertension and know that they are suffering from hypertension [17].

Cholesterol is a risk factor that can be changed from hypertension, so the higher the total cholesterol level, the higher the likelihood of hypertension [18]. The constriction and the rigidity of the blood vessel walls resulting from the buildup of cholesterol in the blood vessels that can cause increased blood pressure will have an impact on the increased risk of CHD. High cholesterol levels in the blood cause cholesterol deposits on blood vessel walls or the so-called plaque cholesterol. The precipitation of calcium ions in plaque cholesterol causes the soft plaque to become hard and rigid. This causes the blood vessel wall to become stiff and not elastic. In addition, in the presence of a hardened plaque cholesterol, this causes the inner walls of blood vessels to become narrow and not slippery, so that blood supply to the organ becomes reduced. If hardening occurs in the arteries that supply blood to the heart (coronary artery), then it causes CHD [19].

In addition, this study is also in line with the theory that the increase in fat levels is associated with the process of atherosclerosis. Dyslipidemia is an important risk factor for the initiation and progression of atherosclerosis and is strongly associated with cardiovascular events [20]. Pathophysiology of CHD originated from the formation of atherosclerosis [21]. Atherosclerosis is the formation of plaque in the walls of the large arteries, thus narrowing the lumen of the vessels that cause the blood flow disruption and decreases the elasticity of the blood vessels. Various studies have been conducted suspecting that the early lesions of atherosclerosis form a layer of fat. Dyslipidemia is a lipid metabolic disorder characterized by increased or decreased lipid fraction in plasma. The major lipid fraction disorders are increased total cholesterol, LDL cholesterol, triglycerides, and decreased HDL cholesterol levels. All lipid fractions have an important role in the process of atherosclerosis and are closely related to one another.

High triglyceride levels and high LDL cholesterol and low HDL cholesterol levels are associated with atherosclerosis, which is one of the risk factors for CHD. The results of Iskandar's study (2017) showed that there was a correlation between triglyceride cholesterol level and CHD occurrence, where the value of OR 1.99 (95% CI 0.97-1.00) was obtained, meaning that patients with high triglyceride levels had odds for CHD 1.99 times greater than patients who have normal triglyceride levels [22]. This is also consistent with Bao et al.'s study which found that total cholesterol, LDL cholesterol, HDL, and triglyceride levels were on average higher in patients with CHD than in the non-CHD group.

Increased total cholesterol levels in the blood leads to cholesterol deposits in the walls of blood vessels. In addition, the increase in total cholesterol also causes disruption to endothelial function by increasing the production of oxygen free radicals. This radical deactivates the production of nitric oxide, a major endothelial-relaxing factor. So if there is an increase in total cholesterol levels and increased levels of triglycerides in a long time, the endothelial permeability becomes increased which causes lipoproteins accumulation in it. Exposure of free radicals in endothelial cell endothelial cells causes LDL oxidation.

Previous research conducted in Korea showed that there was a strong relationship between LDL cholesterol and increased risk of CHD [23]. Low Density Lipoprotein (LDL) is the primary lipid in the process of formation of atherosclerosis. Based on the Guidelines for the Management of Dyslipidemia issued by the Indonesian Heart Association (IHA) in 2013, there is strong evidence of a link between LDL cholesterol and CHD occurrence based on clinical outcome studies, so that LDL cholesterol is the primary target in the management of dyslipidemia. The process of atherosclerosis begins with endothelial damage or dysfunction in artery walls. The possible cause of this endothelial damage can be caused by increased LDL levels. When LDL levels are high, then cholesterol elevated by LDL may precipitate in the subendothelial layer; therefore, LDL is atherogenic, that is the material that can cause atherosclerosis. When the endothelium presents a lesion, oxidized LDL causes various inflammatory reactions, which eventually attract monocytes and neutrophils into the lesion area and increase the size of atheromatous plaque. It will also get worse if followed by a decrease in HDL levels.

Previous research conducted in Cameroon, Central Africa, showed that decreased HDL levels are the most common lipid lesions in causing CHD [21]. High Density Lipoprotein (HDL) is a lipid that acts as a protective factor. HDL plays an important role in reverse cholesterol traction (RCT), a process whereby excess cholesterol in peripheral tissue is returned to the liver for excretion. This process is often referred to as the main mechanism of HDL that is to protect the body from the risk of atherosclerosis and can even decrease the plaque regression. If there is a decrease in HDL levels, then the body protective against atherosclerosis will be reduced; consequently, the inner wall of blood vessels becomes narrow, not slippery, and not elastic, so that the blood supply to the organ becomes reduced. If the process continues on coronary arteries, it will cause coronary heart disease [24].

The results of this study indicated that there was no significant relationship between age and the incidence of CHD, but age was confounder of dyslipidemia relationship with CHD events. The results of this study differ from previous studies which stated that age is significantly associated with the incidence of CHD [25].

## 5. Conclusion

In the CHD group, the percentage of respondents with dyslipidemia was 50%, while in the non-CHD group, the percentage of respondents with dyslipidemia was 17.3%. The relationship of dyslipidemia with CHD differed according to hypertensive status of respondents. Once controlled for age, in hypertensive respondents or having a history of hypertension, respondents with dyslipidemia are 18 times higher to develop CHD than nondyslipidemic respondents. Whereas in respondents who are not hypertensive or have no history of hypertension, respondents with dyslipidemia are 2.5 times higher to develop CHD than nondyslipidemic respondents.

## Figures and Tables

**Table 1:** Relationship of dyslipidemia and covariates with coronary heart disease.

Variables	Control (%)	Case (%)	OR	95% CI
Dyslipidemia				
(i) No	82.7	50.0		
(ii) Yes	17.3	50.0	4.8	2.2 – 10.3
Age				
(i) < 60 years	74.1	67.1		
(ii) ≥ 60 years	25.9	32.9	1.4	0.7 – 2.8
Gender				
(i) Female	54.3	25.6		
(ii) Male	45.7	74.4	3.5	1.7 – 6.9
Family history of CHD				
(i) No	86.4	75.6		
(ii) Yes	13.6	24.4	2.1	0.9 – 4.7
Smoking habit				
(i) No	66.7	53.7		
(ii) Yes	33.3	46.3	1.7	0.9 – 3.3
Hypertension or Hypertension History				
(i) No	75.3	62.2		
(ii) Yes	24.7	37.8	1.9	0.9 – 3.7
Diabetes or Diabetes history				
(i) No	53.1	46.3		
(ii) Yes	46.9	53.7	1.3	0.7 – 2.4
Body Mass Index				
(i) Nonobese	59.3	62.2		
(ii) Obese	40.7	37.8	0.9	0.5 – 1.7

**Table 2:** Description of age and blood pressure in CHD and non-CHD groups.

Variables	N	Mean	SD	Min – Max
Age				
(i) CHD group	82	56.5	9.5	37 – 83
(ii) Non-CHD group	81	51.5	11.3	21 – 76
Systolic blood pressure (SBP)				
(i) CHD group	82	126.2	21.0	100 – 200
(ii) Non-CHD group	81	115.2	20.4	90 – 155
Diastolic blood pressure (DBP)				
(i) CHD group	82	85.6	15.5	60 – 120
(ii) Non-CHD group	81	80.5	14.0	60 – 110

**Table 3:** Full model of dyslipidemia with CHD.

Variables	B	*p* value	OR	CI 95%
Dyslipidemia	1.000	0.049	2.7	1.0– 7.4
Hypertension	-0.29	0.194	0.5	0.2– 1.4
Age ≥ 60 years	0.621	0.128	1.9	0.8– 4.1
Male	1.275	0.002	3.6	1.6– 8.2
Having family history of CHD	0.505	0.295	1.7	0.6– 4.3
Smoker	-0.037	0.926	1.0	0.4– 2.1
Diabetes	0.081	0.824	1.1	0.5– 2.2
Obese	-0.243	0.512	0.8	0.4– 1.6
Dyslipidemia*∗*Hypertension	1.877	0.038	6.5	1.1 – 38.6

**Table 4:** Final model of dyslipidemia connection with CHD.

Variables	B	*p* value	OR	CI 95%
Dyslipidemia	0.924	0.047	2.5	1.0 – 6.3
Hypertension	0.545	0.150	1.7	0.8 – 3.6
Age ≥ 60 years	-0.642	0.227	0.5	0.2 – 1.5
Dyslipidemia*∗*Hypertension	1.973	0.022	7.2	1.3 – 38.7

**Table 5:** Relationship of dyslipidemia with coronary heart disease according to hypertension.

	*p* value	OR	CI 95%
Dyslipidemia in nonhypertension group	0.047	2.5	1.0– 6.3
Dyslipidemia in hypertension group	<0.001	18.1	4.3 – 75.6

## Data Availability

The data used to support the findings of this study are available from the corresponding author upon request.

## Additional Points

*Recommendation*. Health workers should prioritize preventive programs by improving communication, information, and education to the public regarding lifestyles, especially those related to positive changes in behavior to control lipid profile and blood pressure. Changing lifestyles can be done by dieting healthy foods such as reducing saturated fat intake, increasing fiber intake, and reducing carbohydrate intake to control lipid profile and blood pressure and regular medical checkup in Posbindu Noncommunicable Diseases or Health Service Facilities.

## References

[1] World Health Organization (2014). *Global Status Report on Non-Communicable Diseases 2014*.

[2] Gupta R., Mohan I., Narula J. (2016). Trends in coronary heartd disease epidemiology in india. *Annals of Global Health*.

[3] Zhao M., Vaartjes I., Graham I. (2017). Sex differences in risk factor management of coronary heart disease across three regions. *Heart*.

[4] Kementerian Kesehatan RI (2014). *Situasi Kesehatan Jantung*.

[5] Kementerian Kesehatan RI (2013). *Laporan Riset Kesehatan Dasar (RISKESADAS) 2013*.

[6] Melander O. (2015). Genetics of coronary heart disease: towards causal mechanism, novel drug targets and more personalized prevention. *Journal of Internal Medicine*.

[7] Nelson R. H. (2013). Hyperlipidemia as a risk factor for cardiovascular disease. *Primary Care: Clinics in Office Practice*.

[8] Lin C.-F., Chang Y.-H., Chien S.-C., Lin Y.-H., Yeh H.-Y. (2018). Epidemiology of dyslipidemia in the asia pacific region. *International Journal of Gerontology*.

[9] Perkumpulan Endokrinologi Indonesia (PERKENI) (2015). *Konsensus Pengelolaan Dislipidemia di Indonesia*.

[10] Imano H., Noda H., Kitamura A. (2011). Low-density lipoprotein cholesterol and risk of coronary heart disease among japanese men and women: the circulatory risk in communities study (CIRCS). *Preventive Medicine*.

[11] Djeric M., Stokic E., Vuckovic B., Kojic-Damjanov S., Cabarkapa V. (2006). Lipids and atherosclerosis. *Jugoslovenska medicinska biohemija*.

[12] Garg R., Aggarwal S., Kumar R., Sharma G. (2015). Association of atherosclerosis with dyslipidemia and co-morbid conditions: A descriptive study. *Journal of Natural Science, Biology and Medicine*.

[13] Kabakci G., Koylan N., Ilerigelen B. (2008). Impact of dyslipidemia on cardiovascular risk stratification of hypertensive patients and association of lipid profile with other cardiovascular risk factors: results from the ICEBERG study. *Integrated blood pressure control*.

[14] de Rojas F. D., De Frutos T., Ponte A., Chacón J. M., Vitale G. C. (2009). Coronary heart disease and dyslipidemia: a cross-sectional evaluation of prevalence, current treatment, and clinical control in a large cohort of Spanish high-risk patients: the PRINCEPS study. *Preventive Cardiology*.

[15] Rusmi R., Oemiati R. (2014). Penyakit jantung koroner dengan obesitas di kelurahan kebon kalapa bogor. *Buletin Penelitian Sistem Kesehatan*.

[16] Dalal J. J., Padmanabhan T. N., Jain P., Patil S., Vasnawala H., Gulati A. (2012). Lipitension: Interplay between dyslipidemia and hypertension. *Indian Journal of Endocrinology and Metabolism*.

[17] Astiari N. P. T. (2016). *Faktor-Faktor yang Mempengaruhi Kejadian Hipertensi pada Laki-Laki Dewasa di Puskesmas Payangan Kecamatan Payangan Kabupaten Gianya*.

[18] Fujikawa S., Iguchi R., Noguchi T. (2015). Cholesterol crystal embolization following urinary diversion: a case report. *Hinyokika Kiyo: Acta Urologica Japonica*.

[19] Supriyono M. (2008). *Faktor- Faktor Risiko yang Berpengaruh Terhadap Kejadian Penyakit Jantung Koroner pada Kelompok Usia < 45 Tahun di RSUP Kariadi dan RS Telogorejo Semarang*.

[20] Bittner V. (2005). Perspectives on dyslipidemia and coronary heart disease in women. *Journal of the American College of Cardiology*.

[21] Schaefer E. J. (2010). *High Density Lipoproteins, Dyslipidemia, And Coronary Heart Disease*.

[22] Iskandar I., Hadi A., Alfridsyah A. (2017). Faktor Risiko Terjadinya Penyakit Jantung Koroner pada Pasien Rumah Sakit Umum Meuraxa Banda Aceh. *AcTion: Aceh Nutrition Journal*.

[23] Boo S., Froelicher E. S. (2012). Cardiovascular risk factors and 10-year risk for coronary heart disease in Korean women. *Asian Nursing Research*.

[24] Ama Moor V. J., Ndongo Amougou S., Ombotto S., Ntone F., Wouamba D. E., Ngo Nonga B. (2017). Dyslipidemia in patients with a cardiovascular risk and disease at the university teaching hospital of Yaoundé, Cameroon. *International Journal of Vascular Medicine*.

[25] Setyoko, Anggraini M. T., Huda U. (2012). Dislipidemia Sebagai Faktor Risiko Penyakit Jantung iskemik di RSUD Tugurejo Semarang. *Semarang*.

